# Nintedanib Induces Mesenchymal-to-Epithelial Transition and Reduces Subretinal Fibrosis Through Metabolic Reprogramming

**DOI:** 10.3390/ijms26157131

**Published:** 2025-07-24

**Authors:** David Hughes, Jüergen Prestle, Nina Zippel, Sarah McFetridge, Manon Szczepan, Heike Neubauer, Heping Xu, Mei Chen

**Affiliations:** 1 The Wellcome-Wolfson Institute for Experimental Medicine, Belfast BT9 7BL, Northern Ireland, UK; david.hughes@qub.ac.uk (D.H.); manon.szczepan@childrens.harvard.edu (M.S.);; 2 Boehringer Ingelheim Pharma GmbH & Co. KG, 88400 Biberach Riss, Germany; juergen.prestle@boehringer-ingelheim.com (J.P.); heike.neubauer@boehringer-ingelheim.com (H.N.)

**Keywords:** age-related macular degeneration, retinal pigment epithelial (RPE) cell, macular fibrosis, epithelial to mesenchymal transition (EMT), mesenchymal to epithelial transition (MET)

## Abstract

This study aimed to investigate the tyrosine kinase inhibitor Nintedanib and its potential role in reversing epithelial–mesenchymal transition (EMT) induced by transforming growth factor beta 2 (TGF-β2) in retinal pigment epithelial (RPE) cells, along with its therapeutic potential using a mouse model of subretinal fibrosis. We hypothesized that the blockade of angiogenesis promoting and fibrosis inducing signaling using the receptor tyrosine kinase inhibitor Nintedanib (OfevTM) can prevent or reverse EMT both in vitro and in our in vivo model of subretinal fibrosis. Primary human retinal pigment epithelial cells (phRPE) and adult retinal pigment epithelial cell line (ARPE-19) cells were treated with TGF-β210 ng/mL for two days followed by four days of Nintedanib (1 µM) incubation. Epithelial and mesenchymal phenotypes were assessed by morphological examination, quantitative real-time polymerase chain reaction(qPCR) (*ZO-1*, *Acta2*, *FN*, and *Vim*), and immunocytochemistry (ZO-1, vimentin, fibronectin, and αSMA). Metabolites were measured using luciferase-based assays. Extracellular acidification and oxygen consumption rates were measured using the Seahorse XF system. Metabolic-related genes (*GLUT1*, *HK2*, *PFKFB3*, *CS*, *LDHA*, *LDHB*) were evaluated by qPCR. A model of subretinal fibrosis using the two-stage laser-induced method in *C57BL/6J* mice assessed Nintedanib’s therapeutic potential. Fibro-vascular lesions were examined 10 days later via fluorescence angiography and immunohistochemistry. Both primary and ARPE-19 RPE stimulated with TGF-β2 upregulated expression of fibronectin, αSMA, and vimentin, and downregulation of ZO-1, consistent with morphological changes (i.e., elongation). Glucose consumption, lactate production, and glycolytic reserve were significantly increased in TGF-β2-treated cells, with upregulation of glycolysis-related genes (*GLUT1*, *HK2*, *PFKFB3*, *CS*). Nintedanib treatment reversed TGF-β2-induced EMT signatures, down-regulated glycolytic-related genes, and normalized glycolysis. Nintedanib intravitreal injection significantly reduced collagen-1+ fibrotic lesion size and Isolectin B4+ neovascularization and reduced vascular leakage in the two-stage laser-induced model of subretinal fibrosis. Nintedanib can induce Mesenchymal-to-Epithelial Transition (MET) in RPE cells and reduce subretinal fibrosis through metabolic reprogramming. Nintedanib can therefore potentially be repurposed to treat retinal fibrosis.

## 1. Introduction

Age-related macular degeneration (AMD) represents a significant public health challenge for individuals in the western world aged 60 years of age and older, accounting for approximately 9% of global cases [1]. It is estimated that 288 million people will be affected by this irreversible condition by the year 2040 due to the increase in aging populations worldwide [2]. Advanced AMD manifests in two forms: geographic atrophy (GA) and neovascular AMD (nAMD), with the latter being the major cause of AMD-related blindness [1,2]. nAMD is characterized by the pathological ingrowth of aberrant blood vessels into the macula, with intravitreal injection administration of vascular endothelial growth factor (VEGF) inhibitors serving as the established standard of care [3,4,5,6]. However, nearly half of the patients continue progressing to end-stage blindness primarily as a result of macular fibrosis [3,4,5,7,8,9,10]. At present, no medications exist which prevent or treat the condition and novel strategies, based on the disease mechanism, are urgently needed.

The nAMD-mediated macular fibrosis is a fibro-vascular membrane [7] and fibrotic and angiogenic components of the lesion can be imaged clinically by optical coherence tomography (OCT) and OCT angiography (OCTA) [11,12,13]. Histological investigations showed that the lesion contains not only blood vessels and myofibroblasts but also many infiltrating immune cells including macrophages and T/B cells suggesting the inflammatory nature of the lesion [14]. We previously showed that the complement system contributes critically to macular fibrosis [15,16]. Patients with macular fibrosis have been shown to have higher levels of lipocalin-2 in plasma [17] and increased intraocular levels of soluble intercellular adhesion molecule-1 (ICAM-1) [18]. The ICAM-1/VLA4 pathway can contribute to the development of macular fibrosis by facilitating immune cell recruitment, migration, and activation of cells including macrophages [18]. These results highlight the inflammation role in the development of macular fibrosis.

The macular inflammatory microenvironment can induce fibrosis in choroidal neovascularization (CNV) through the recruitment of fibrocytes [19], and the induction of myofibroblast trans-differentiation from other cells, including retinal pigment epithelial (RPE) cells, vascular endothelial cells, Müller cells, and infiltrating macrophages [7]. A fundamental process in nAMD progression is the epithelial-to-mesenchymal transition (EMT), whereby RPE cells lose their epithelial characteristics and adopt mesenchymal traits, such as enhanced migratory and proliferation capacity [15,20]. It has been demonstrated that growth factors, including fibroblast growth factor-2 (FGF-2), transforming growth factor beta (TGF-β), platelet-derived growth factor (PDGF), and cytokine tumor necrosis factor-α (TNF-α) can stimulate EMT in RPE cells [15,20,21].

The tyrosine kinase inhibitor (TKI), Nintedanib, inhibits multiple receptor tyrosine kinases (RTKs), including platelet-derived growth factor receptors (PDGFR), fibroblast growth factor receptors (FGFR), and vascular endothelial growth factor receptors (VEGFR) [22,23,24,25,26]. Nintedanib is used to treat idiopathic pulmonary fibrosis (IPF) [27] and systemic sclerosis-associated interstitial lung disease (SSc-ILD) [23,28]. Previous studies have shown that Nintedanib can inhibit EMT in alveolar epithelial cells [29,30], lung cancer cells [31], and RPE cells [32]. Here we investigated whether Nintedanib can reverse EMT in transforming growth factor beta 2 (TGF-β2) treated RPE cells and the underlying mechanisms. We further explored its therapeutic potential in an in vivo model of subretinal fibrosis.

## 2. Results

### 2.1. Nintedanib Reversed Transforming Growth Factor Beta 2 (TGF-β2) Induced Morphological Change and Reduced Proliferation in Retinal Pigment Epithelial (RPE) Cells

**Figure 1:**
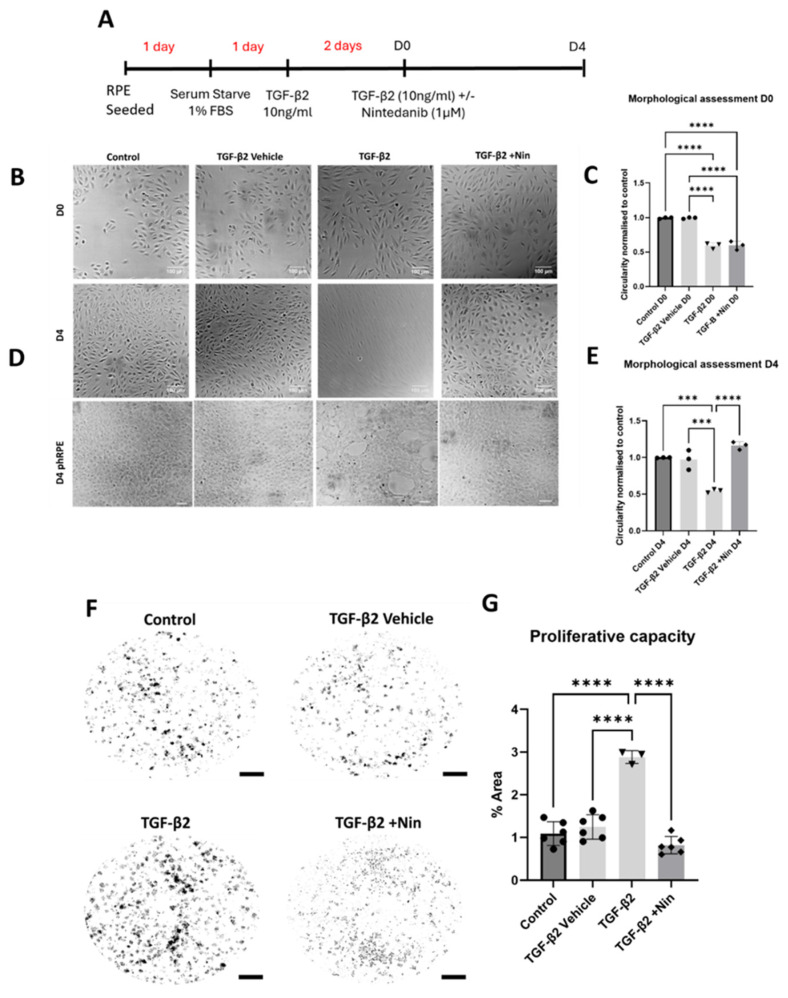
The effect of Nintedanib on transforming growth factor beta 2 (TGF-β2) induced retinal pigment epithelial (RPE) cell proliferation and morphological alteration. (**A**) Schematic diagram showing the experimental design. Treatment time before Nintedanib and establishing epithelial mesenchymal transition (EMT) are highlighted in red, and time is displayed in days. From Nintedanib treatment and assessments time is represented in black (D0-D4). Both the adult retinal pigment epithelial cell line (ARPE-19) and primary human retinal pigment epithelial (phRPE) cells were treated with 10 ng/mL of TGF-β2 for 48 h followed by an additional 96 h of Nintedanib treatment. (**B**) Representative phase-contrast images from different groups showing the morphology of ARPE19 cells at D0 (immediately before Nintedanib treatment). (**C**) Normalized cell circularity index in cells from different groups at D0. Scale bar = 100 µm. Data is representative of 3 repeated studies n = 10 technical repeats per group/study for ARPE-19 cells. Individual data points and mean ± SD are shown. **** *p* < 0.0001. One-way ANOVA, with Tukey’s post hoc analysis. (**D**) Representative phase-contrast images from different groups showing the morphology of ARPE-19 cells and phRPE cells at D4 after Nintedanib treatment, n = 10 technical repeats per group/study for ARPE-19 cells, n = 3 human donors for phRPE cells. (**E**) Normalized cell circularity index in cells from different groups at D4. Scale bar = 100 µm. Data is representative of 3 repeated studies, n = 10 technical repeats per group/study for ARPE-19 cells. Individual data points and mean ± SD are shown. *** *p* < 0.001; **** *p* < 0.0001. One-way ANOVA, with Tukey’s post hoc analysis. (**F**) Representative images showing phRPE cell colony formation in different groups. Scale bar = 4 mm. (**G**) Bar figure showing the changes in colony area in different treatment groups compared to the control group. Data is representative of 3–6 different human donors, with 3 technical repeats per donor. Individual data points and mean ± SD are shown. **** *p* < 0.0001. One-way ANOVA, with Tukey’s post hoc analysis.

To investigate whether Nintedanib can reverse EMT, RPE cells were treated with transforming growth factor beta 2 (TGF-β2) for two days (day 0, D0) before Nintedanib exposure (Figure 1A) to ensure a fully established EMT state. One day after TGF-β2 stimulation, adult retinal pigment epithelial cell line (ARPE-19) cells began to exhibit elongated and spindle-like morphology along with significantly reduced circularity indexes characteristic of EMT, which persisted from D0 (Figure 1C) to D4 of observation (Figure 1B). Nintedanib treatment resulted in a notable reversal of the TGF-β2-induced morphological changes, exhibiting a transition from the elongated shape back to cobblestone morphology observed in control cells with significantly higher circularities. This reversal in cell morphology was observed from D2 and became more pronounced over time until D4 (Figure 1B,C). Primary human retinal pigment epithelial cells (phRPE) at D4 (Figure 1D) also showed noticeable morphological differences including large vacuole-like spaces developing between groups of cells. phRPE treated with Nintedanib also exhibited reversed EMT characteristics with morphology matching that of control and vehicle treatment groups. Dose responsive effects of Nintedanib along with responsiveness on mesenchymal-to-epithelial transition (MET) over time (D0-D4) were also observed (Supplementary Figure S1).

In a clonogenic functional assessment following the same experimental setup, phRPE treated with TGF-β2 had a significant increase in colony formation compared to that of control and vehicle-treated cells and this was reversed by Nintedanib (Figure 1F,G). The number of colonies formed by cells treated with Nintedanib was comparable to that of control and vehicle-treated cells.

### 2.2. Nintedanib Restored Epithelial Markers and Reduced Epithelial Mesenchymal Transition (EMT) Markers in Transforming Growth Factor Beta 2 (TGF-β2) Treated Retinal Pigment Epithelial (RPE) Cells

**Figure 2:**
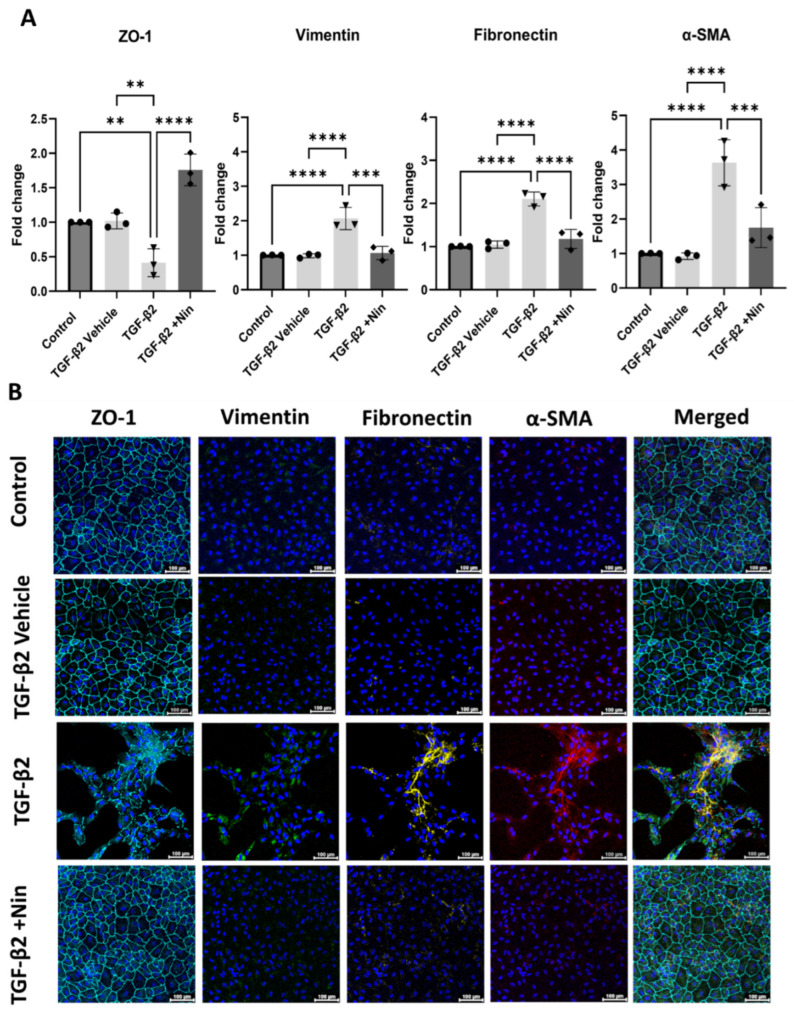
Effect of Nintedanib on transforming growth factor beta 2 (TGF-β2) induced epithelial-to-mesenchymal transition in primary human retinal pigment epithelial (phRPE) cells. The phRPE were treated with TGF-β2 (10 ng/mL) for 48 h followed by 4 days of Nintedanib (1 µM) treatment. (**A**) quantitative polymerase chain reaction (qPCR) analysis of zonula occludens -1 (*ZO-1*), fibronectin (*Fn*), vimentin (*Vim*), and alpha smooth muscle actin (*Acta2*) genes. Individual data points and mean ± SD are shown. n = 3 biological human donors, with 3 technical repeats per donor. ** *p* < 0.01; *** *p* < 0.001; **** *p* < 0.0001. One-way ANOVA, with Tukey’s post hoc analysis. (**B**) Representative immunocytochemistry images for ZO-1, vimentin, fibronectin, and αSMA in different groups. Scale bar 100 µm. n = 3 human donors, with 3 technical repeats per donor. Blue; Dapi; Cyan; ZO-1, Green; Vimentin; Yellow; Fibronectin, Red; α-SMA.

Quantitative PCR (qPCR) revealed a significant downregulation of epithelial marker Zonula occludens-1 (*ZO-1*) but an upregulation of Vimentin (*Vim*), Fibronectin (*FN*), and alpha-smooth muscle actin (*Acta2*)genes compared to control and vehicle-treated cells (Figure 2A). However, treatment with Nintedanib significantly upregulated *ZO-1* and down-regulated *Vim*, *FN*, and *Acta2* genes in TGF-β2-treated cells (Figure 2A). Immunocytochemistry confirmed the downregulation of ZO-1 and upregulation of vimentin, fibronectin, αSMA in TGF-β2-treated cells (Figure 2B) and this was reversed by Nintedanib treatment (Figure 2B), accompanied by morphological changes described above in Figure 1. Taken together, these results suggest that Nintedanib effectively reversed TGF-β2-induced EMT in phRPE cells at the molecular level, promoting the maintenance of epithelial characteristics, and highlighting a potential therapeutic utility in preserving epithelial integrity in pathologies associated with EMT dysregulation. Results were confirmed in ARPE-19 (Supplementary Figure S2).

### 2.3. The Effect of Nintedanib on Metabolic Regulation in Transforming Growth Factor Beta 2 (TGF-β2) Treated Retinal Pigment Epithelial (RPE) Cells

**Figure 3:**
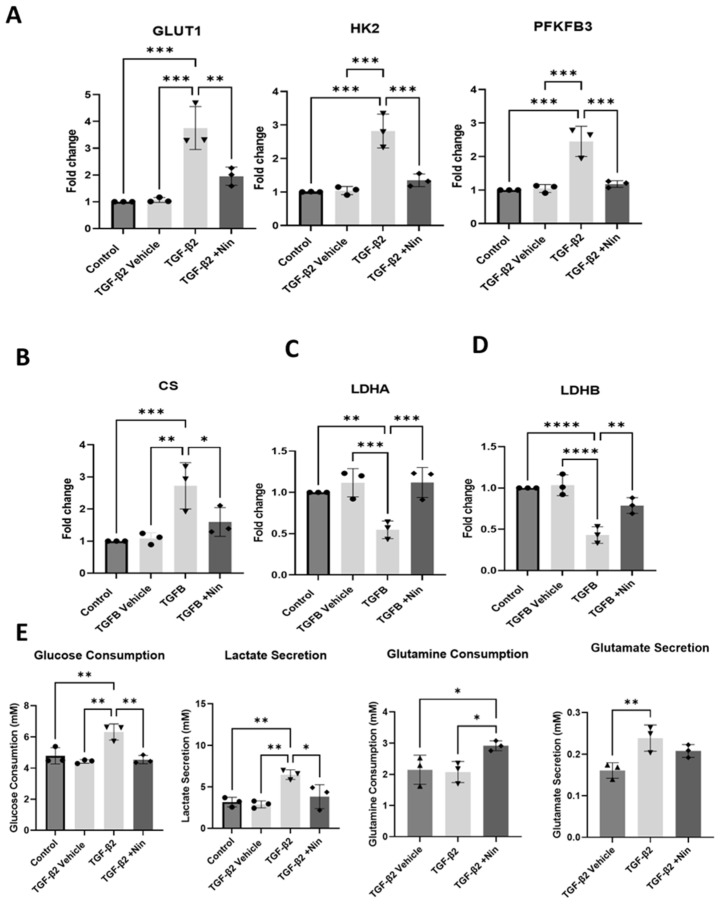
Effect of Nintedanib on transforming growth factor beta 2 (TGF-β2) induced metabolic alteration in primary human retinal pigment epithelial (phRPE) cells. phRPE were treated with TGF-β2 (10 ng/mL) for 48 h followed by 4 days of Nintedanib (1 µM) treatment. (**A**) The expression of glucose transporter 1 (*GLUT1*), hexokinase 2 (*HK2*), and 6-phosphofructo-2-kinase/fructose-2,6-bisphosphatase 3 (*PFKFB3*) genes in different groups assessed by quantitative polymerase chain reaction (qPCR). Individual data points and mean ± SD are shown. n = 3 biological human donors, with 3 technical repeats per donor. ** *p* < 0.01; *** *p* < 0.001. One-way ANOVA, with Tukey’s post hoc analysis. (**B**–**D**) The expression of citrate synthase (*CS*), lactate dehydrogenase A and B (*LDHA*, and *LDHB*) genes in different groups assessed by qPCR. Individual data points and mean ± SD are shown. n = 3 human donors, with 3 technical repeats per donor. * *p* < 0.05; ** *p* < 0.01; *** *p* < 0.001; **** *p* < 0.0001. One-way ANOVA, with Tukey’s post hoc analysis. (**E**) Glucose and glutamine consumption, and lactate and glutamate secretion in different groups measured by Luciferase assay. Individual data points and mean ± SD are shown. n = 3 biological human donors, with 3 technical repeats per donor. * *p* < 0.05; ** *p* < 0.01. One-way ANOVA, with Tukey’s post hoc analysis.

Upon TGF-β2 stimulation, the expression of glycolytic genes including glucose transporter 1 (*GLUT1*), hexokinase 2 (*HK2*), and 6-phosphofructo-2-kinase/fructose-2,6-bisphosphatase 3 (*PFKFB3*) was significantly elevated (Figure 3A). Nintedanib treatment reversed TGF-β2-induced upregulation of *GLUT1*, *HK2*, and *PFKFB3* (Figure 3A). The expression of citrate synthase (*CS*), a key enzyme in the tricarboxylic acid (TCA) cycle, remained elevated in both TGF-β alone and TGF-β+ Nintedanib-treated cells, however a downward trend was observed in the Nintedanib treated cells (Figure 3B). The expression levels of lactate dehydrogenase A (*LDHA*) and lactate dehydrogenase B (*LDHB*) were both significantly reduced in TGF-β2 treated cells (Figure 3C,D). Nintedanib treatment reversed *LDHA* but not *LDHB* expression (Figure 3C,D).

In line with the mRNA expression of metabolic-related genes, TGF-β2-treated cells consumed a significantly higher amount of glucose and produced more lactate compared to control and vehicle-treated cells (Figure 3E). Whereas glutamine consumption was not significantly altered between TGF-β2-treated cells and control or vehicle-treated cells, glutamate production was shown to increase after TGF-β2 treatment. The addition of Nintedanib to TGF-β2-treated cells resulted in a significantly reduced glucose consumption and lactate secretion compared to cells treated with TGF-β2 alone. The glutamine consumption was significantly increased in Nintedanib-treated cells compared to TGF-β2-treated cells.

**Figure 4:**
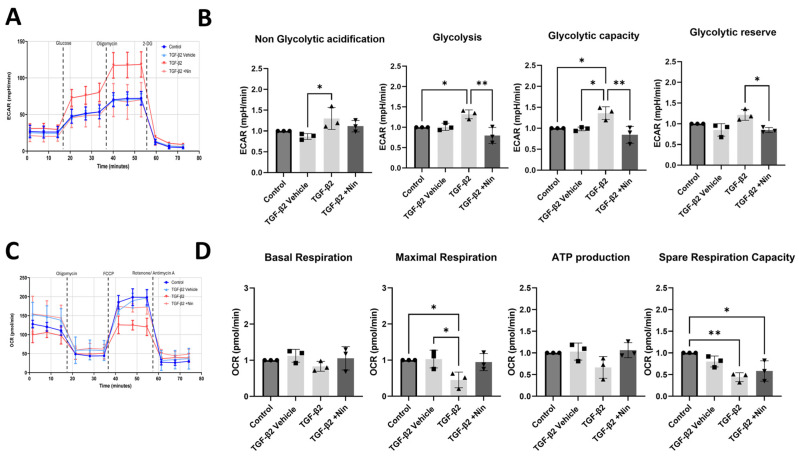
The effect of transforming growth factor beta 2 (TGF-β2) and Nintedanib on primary human retinal pigment epithelial (phRPE) cell glycolysis and oxidative phosphorylation. phRPE were treated TGF-β2 (10 ng/mL) for 48 h followed by Nintedanib (1 µM) for an additional 96 h. The extracellular acidification rate (ECAR) and oxygen consumption rate (OCR) were measured using the Seahorse XFe96 system following the manufacturer’s instructions. (**A**) Representative image showing glycolysis based on ECAR measurements in different groups. (**B**) Bar figures showing non-glycolytic acidification, glycolysis, glycolytic capacity, and glycolytic reserve. * *p* < 0.05, ** *p* < 0.01. One-way ANOVA, with Tukey’s post hoc analysis. Individual data points and mean ± SD are shown. Data is representative of n = 3 biological human donors, with 10 technical replicates per group. (**C**) Representative image showing mitochondrial oxidative phosphorylation based on OCR measurements in different groups. (**D**) Bar figures show mitochondrial basal respiration, maximal respiration, ATP production, and spare respiration capacity. * *p* < 0.05, ** *p* < 0.01. One-way ANOVA, with Tukey’s post hoc analysis. Individual data points and mean ± SD are shown. Data is representative of n = 3 human donors, with 10 technical replicates per group.

The real-time metabolic profile was further tested using the Seahorse XFe96 system. In this assay, glycolysis and mitochondrial oxidative phosphorylation were assessed by measuring the extracellular acidification rate (ECAR) and oxygen consumption rate (OCR), respectively. TGF-β2-treated cells exhibited a higher level of glycolytic capacity and glycolytic reserve compared to control cells (Figure 4A). Treatment with Nintedanib reversed TGF-β2-induced upregulation of glycolysis, glycolytic capacity and glycolytic reserve (Figure 4B). The OCR analysis showed that TGF-β2-treated cells exhibited a reduced spare respiratory capacity compared to control cells, and a reduced maximal respiration indicating a compromised ability to respond to increased energy demands or stress conditions (Figure 4C). Nintedanib treatment did not significantly affect TGF-β2-induced reduction in spare respiratory capacity (Figure 4D).

Taken together, our results suggest that TGF-β2 drives a metabolic shift towards aerobic glycolysis in phRPE cells during EMT and this was reversed by Nintedanib. Furthermore, Nintedanib can also shift towards glutamine utilization as an alternative metabolic fuel source in TGF-β2-induced myofibroblasts.

### 2.4. Nintedanib Reduces Fibrotic Lesions in the Mouse Model of Two-Stage Laser-Induced Subretinal Fibrosis

**Figure 5:**
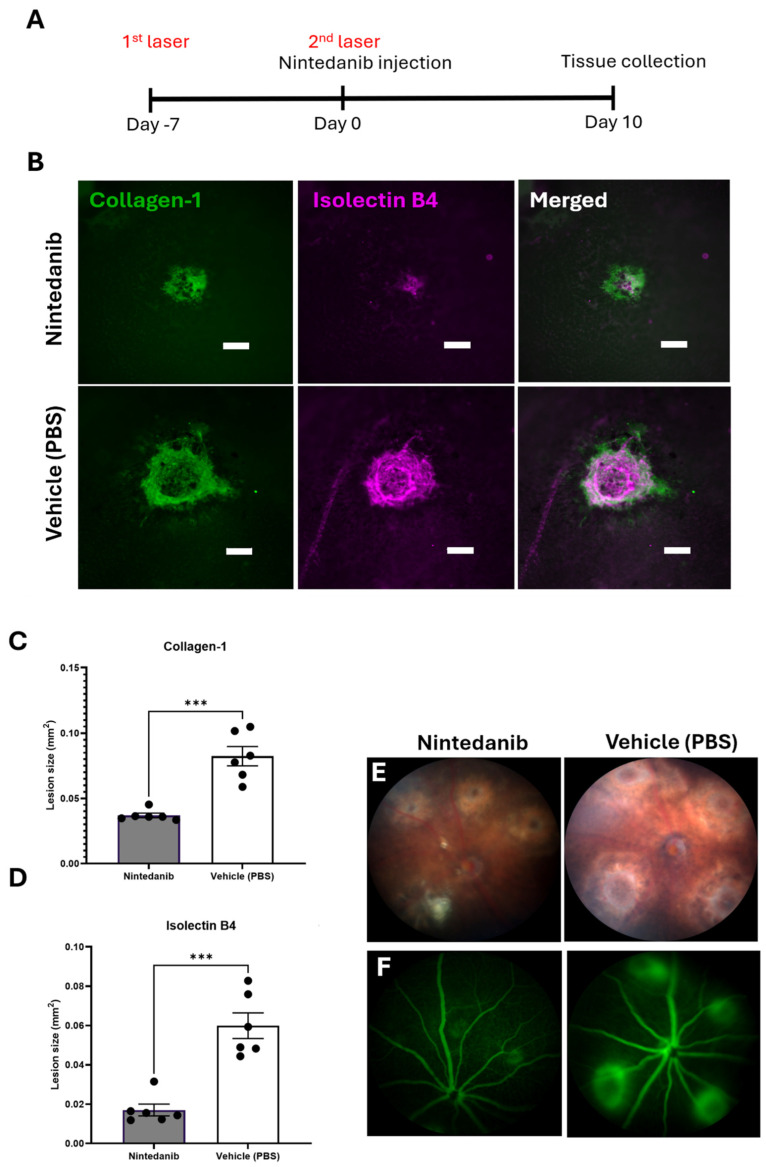
Effect of Nintedanib on a mouse model of subretinal fibrosis. (**A**) Schematic diagram detailing the experimental design. Subretinal fibrosis was induced in *C57BL/6J* mice using a two-stage laser-induced protocol. Nintedanib (12 μg/eye/μL) or vehicle control (PBS + 0.015% Tween 80, 1 μL/eye) was administered by intravitreal injection immediately following the second laser. Fundus imaging and fluorescence angiography (FFA) were conducted 10 days later before animals were culled for tissue collection. (**B**) Representative lesion area, respectively, in Nintedanib and vehicle-treated mice. Scale bar 100 µm (**C**) Quantitative assessment of collagen-1 (**D**) and Isolectin B4. (**E**) Representative fundus imaging of lesions in Nintedanib and vehicle-treated mice at day 10 post-second laser. (**F**) Representative fundus fluorescein angiogram (FFA) images from Nintedanib and vehicle-treated mice at day 10 post-second laser. Scale bar 100 µm. Individual data points and mean ± SD are shown. n = 6 animals per group, (each point represents 8 lesions across two eyes analyzed). *** *p* < 0.001. Two-tailed unpaired *t*-test was used for analysis.

The anti-fibrotic potential of Nintedanib was evaluated in the mouse model of subretinal fibrosis using a two-stage laser protocol developed by us [9]. Nintedanib (12 μg/eye/μL) was injected intravitreally immediately after the second laser. Lesion assessment was performed 10 days later (Figure 5A). Fundus imaging after Nintedanib injection and day 10 of assessment are shown (Supplementary Figure S3). Nintedanib formed whitish/yellowish depositions immediately after intravitreal injection and remained visible under microscopic/slit lamp examinations at the end of the study. RPE/choroidal flat mount staining of collagen type I (Col-1) and Isolectin B4 showed a significant reduction in lesion sizes in the Nintedanib-treated group (Figure 5B). Nintedanib treatment reduced angiogenesis (evidenced by Isolectin B4 staining) by 71.6% and fibrosis (evidenced by Col-1 staining) by 52.5% (Figure 5C,D). Fundus imaging and fluorescein angiography (FFA) also revealed a significant reduction in lesion size and vascular leakage in animals treated with Nintedanib compared to that in animals treated with vehicle (PBS + 0.015% Tween 80) (Figure 5E,F). To further assess the therapeutic potential of Nintedanib, we compared the anti-fibrotic and anti-angiogenic effects of Nintedanib with the highest dose of Aflibercept (40 mg/mL, 40 μg/eye/μL). Our results showed that both Nintedanib and Aflibercept significantly reduced the size of the fibro-vascular lesion, and the reduction in the vascular component of the lesion was more pronounced in Nintedanib-treated mice (Supplementary Figure S4).

## 3. Discussion

Nintedanib has been used to treat idiopathic pulmonary fibrosis since 2014 [27,33]. It has also been used as a second-line therapy for advanced metastatic or recurring non-small cell lung cancer of adenocarcinoma histology [31] due to its strong inhibitory effects on both nonreceptor tyrosine kinases and receptor tyrosine kinases that are involved in the signaling pathways of PDGFR, FGFR, and VEGFR [20,22,23,24,25,26]. In the present study, we show that Nintedanib can reverse TGF-β2-induced EMT in RPE cells and suppress subretinal fibrosis in the two-stage laser-induced mouse model. Mechanistically, we found that Nintedanib could suppress glycolysis in TGF-β2-induced, RPE-originated mesenchymal cells and this metabolic reprogramming-initiated MET.

Aging is one of the prominent risk factors involved in the development of AMD and associated alterations which disrupt retinal health. We showed recently that old age promotes nAMD-mediated subretinal fibrosis [19]. During aging, RPE accumulates cellular damage from oxidative stress and inflammation which promotes pathological properties, such as EMT and metabolic reprogramming. As a result, RPE cells lose epithelial characteristics including cell–cell adhesion properties, ultimately leading to fibrotic scar formation in the late stages of the disease. In addition, the diseased microenvironment triggers a metabolic shift, further destabilizes RPE and promotes increased EMT [3,7,19,34]. The role of metabolic reprogramming in cell activation and differentiation is well acknowledged. During EMT, the mesenchymal cells utilize glycolysis as a major source of energy supply to fulfill the demands for high levels of extracellular matrix production [35,36]. We detected high levels of glycolytic-related genes (*GLUT1*, *HK2*, *PFKFB3*) expression, enhanced glucose consumption, and increased lactate secretion in TGF-β2-treated phRPE cells. This is in line with a previous study by Shu et al. [37], whereby the authors reported high levels of glycolysis during EMT in TGF-β2-treated APRE-19 RPE cells. We found the mitochondrial oxidative phosphorylation was also affected in TGF-β2 treated cells (i.e., reduced maximal respiration and spare capacity), which was also observed by Shu et al. with reported mitochondria fragmentation [37]. Shu et al. treated ARPE-19 cells with TGF-β2 for 24 and 72 h, whereas we incubated phRPE cells with TGF-β2 for six days. Furthermore, single cell transcriptomics/proteomics studies have shown altered mitochondrial transcripts and proteins in RPE cells from geographic atrophy patients [34]. Increased expression of genes for oxidative phosphorylation, along with an upregulation of genes and proteins involved in ECM reorganization, support the potential of metabolic reprogramming in driving RPE dysfunction and disease progression [34]

Unlike previous studies whereby the authors treated cells with Nintedanib and TGF-β simultaneously [32], we incubated the cells with TGF-β2 for 2 days before Nintedanib treatment. Previous studies from us [15] and others [37] have shown that 48 h of TGF-β2 treatment is enough to induce EMT in RPE cells. Nintedanib treatment significantly decreased *GLUT1*, *HK2*, and *PFKFB3* gene expression and reduced glycolysis in cells pre-treated with TGF-β2 (Figure 3A). Our results suggest that Nintedanib can reprogram the metabolic pathway by down-regulating key genes involved in glycolysis in mesenchymal cells and initiate MET. MET is important to achieve a functional recovery in the fibrotic organ. This function of Nintedanib may explain its efficiency in advanced stages of IPF [38].

In this study, we used the mouse model of two-stage laser-induced subretinal fibrosis to test the anti-fibrotic potential of Nintedanib. Until now, many studies have used one laser-induced CNV as a model to study subretinal fibrosis. In the traditional laser-induced CNV, the vascular lesion regresses spontaneously within 2–3 weeks although the non-vascular subretinal scar may persist. We previously showed that the two-stage laser protocol resulted in the conversion of the CNV into a large fibro-vascular lesion [9] that mimics closely macular fibrosis in nAMD [7]. Importantly, both the fibrotic and vascular components of the lesion persisted for at least 40 days after the second laser without signs of regression [9]. We found that intravitreal injection of Nintedanib reduced fibrosis and angiogenesis by 52.5% and 71.6%, respectively, in our model. Our results suggest that Nintedanib has great potential to tackle macular fibrosis along with other fibrotic retinal disorders, such as proliferative diabetic retinopathy and proliferative vitreoretinopathy. Future work could include an assessment of mitochondrial morphology after induction of EMT through treatment of TGF-β2 along with cells treated with Nintedanib to assess if fragmentation occurred/the degree and what effect this has on mitochondrial biogenesis.

While this study provides promising insights into Nintedanib’s ability to promote mesenchymal-to-epithelial transition (MET) and reduce fibrosis, certain limitations of the study could be further explored to enhance the findings and support future research. The current findings focus on Nintedanib’s intended therapeutic outcomes; however, given Nintedanib’s role as a broad-spectrum tyrosine kinase inhibitor evaluating possible off-target effects or interactions with unrelated signaling pathways, it could provide a more complete picture of its mechanism of action. Additionally, although Nintedanib effectively reversed glycolytic changes, some mitochondrial oxidative phosphorylation, including spare respiratory capacity and maximal respiration, showed incomplete recovery. This suggests an opportunity to investigate whether extended treatment durations or complementary approaches could fully restore mitochondrial function, further supporting the MET phenotype. Furthermore, the evaluation of Nintedanib’s anti-angiogenic and anti-fibrotic in vivo relied solely on isolectin B4 and Collagen I staining. Additional markers such as VEGF, connective tissue growth factor (CTGF), or fibronectin in future studies could offer a broader perspective on these processes, enhancing the robustness of the findings. Finally, the double-laser CNV model used in this study has been shown to be stable up to day 40. However, this study only examined lesions after a total of 17 days. Extending the study timeline, to show follow up responses may potentially provide further insights into the long-term anti-fibrotic effects of Nintedanib.

Since retinal fibrosis is a localized disorder within the eye, we administered Nintedanib intravitreally in this study. This should be the most effective approach to treat retinal fibrosis. To repurpose Nintedanib for the management of retinal fibrosis, proper formulations will need to be developed.

In conclusion, our study suggests that Nintedanib can induce MET by metabolic reprogramming, i.e., suppressing glycolysis in mesenchymal cells, and Nintedanib may be repurposed to treat fibrotic retinal diseases such as macular fibrosis in nAMD.

## 4. Materials and Methods

### 4.1. Culture and Treatment of RPE Cells

ARPE-19 cells (Cat. CRL-2302, ATCC, Manassas, VA, USA) were cultured in DMEM/F12 media (Cat. 11330032, Thermo Fisher Scientific, Inchinnan, Scotland, UK) and supplemented with 10% FBS (Cat. 10270106, Thermo Fisher Scientific, Inchinnan, Scotland, UK) under standard culture conditions (21% O_2_, 5% CO_2_). A total of 3125 cells/cm^2^ from ARPE-19 culture between passages 4 (P4) to P8 were used in the study. To induce EMT, ARPE-19 cells were seeded in corresponding experimental plates as indicated in each specific experimental methods section for 24 h. The media was replaced with DMEM/F12 + 1% FBS for an additional 24 h (1% FBS was then maintained throughout the entire experiment). The cells were then treated with TGF-β2 (10 ng/mL) for EMT induction (Cat. 7346-B2-005/CF, R&D systems, Minneapolis, MN, USA) or vehicle (4 mM HCl + 0.1% BSA) for two days (Day 0). After that, the cells were treated with Nintedanib (1 µM) for an additional four days (D4) based on initial pilot data and observed morphological changes. Dimethyl sulfoxide (DMSO) was used as vehicle control for Nintedanib.

Primary human RPE (phRPE) (Cat. 6540, ScienCell, Carlsbad, CA, USA) were cultured in plates coated with Poly-l-lysin (Cat.4707-50 mL, Sigma-Aldrich, Gillingham, UK) and epithelial cell medium (EpiCM) (Cat. 4101, ScienCell, Carlsbad, CA, USA) supplemented with 2% FBS (Cat.0010, ScienCell, Carlsbad, CA, USA), Epithelial cell growth supplement (Cat.4152, ScienCell), and penicillin/Streptomycin solution (Cat. 0503, ScienCell, Carlsbad, CA, USA). phRPE were cultured under standard conditions (21% O_2_, 5% CO_2_) and used between passages 1–3. To induce EMT, 8000 cells/cm^2^ of phRPE were seeded in corresponding experimental plates as indicated in each specific experimental method section or 24 h. The media was replaced with EpiCM + 1% FBS for an additional 24 h (1% FBS was then maintained throughout the entire experiment). The cells were then treated with TGF-β2 (10 ng/mL) for EMT induction (Cat. 7346-B2-005/CF, R&D systems, Minneapolis, MN, USA) or vehicle (4 mM HCl + 0.1% BSA) for two days (Day 0). After that, the cells were treated with Nintedanib (1 µM) for an additional four days (D4). DMSO was used as vehicle control for Nintedanib.

### 4.2. Preparation of Nintedanib

Nintedanib stock solution of 10 mM was prepared in 100% DMSO. For all in vitro experiments, this stock solution was used and further diluted in assay media to a final concentration of 1 µM.

For in vivo studies, Nintedanib was prepared as a sustained release suspension formulation by sonification in PBS containing 0.015% Tween 80 for a final concentration of 12 mg/mL. The concentration was determined based on compound solubility and its intended slow-acting release from a crystalline structure. This was validated per microliter to remain active/last for more than 10 days, while remaining viable for injections. Nintedanib was thawed at room temperature 30 min before use and vortexed vigorously to obtain a homogenous solution. The vehicle for Nintedanib in vivo was PBS containing 0.015% Tween 80.

### 4.3. Morphological Assessment

The ARPE-19 cells were imaged on the live cell imager Nikon 6D (Nikon, MI, USA) at both D0 (48 h post-TGF-β2 induction) and D4 (96 h post-TGF-β2). Primary human RPE (phRPE) were imaged in conjunction to validate morphological changes. A total of 50 cells per sample were randomly selected using a grid-based numbering system overlaid on images and analyzed in ImageJ to ensure unbiased quantification. Images were analyzed using ImageJ, and cell area and perimeter were calculated. The circularity of the cells was assessed based on the area and perimeter measured utilizing the formula: circularity = 4 × 3.14 × Area/Perimeter^2^.

### 4.4. Clonogenic Assay

Primary human retinal pigment epithelial (phRPE) cells were seeded in 6-well plates in a total of 2 mL of media containing 100 cells per mL/well and left for 8 days, with treatments following the previously described timeline. Colony formation was analyzed using crystal violet (Cat. C0775, Sigma-Aldrich, Gillingham, UK) which was administered to each well for 1 h at room temperature before excess staining was removed with PBS washes. The plates were additionally submerged in water before being left to dry overnight at room temperature (RT). The entire well of the 6-well plate was then imaged using the EVOS microscope Cell Imaging system (Thermo Fisher Scientific). The analysis of the colonies formed was performed using ImageJ software version 1.53.

### 4.5. RNA Extraction

Total RNA using the Maxwell RSC simplyRNA Cells Kit (Cat. AS1390, Promega, Seattle, WA, USA) was isolated following manufacturer’s instructions. The concentration and purity of RNA was measured by NanoDrop One (Cat. ND-ONE-W, Thermo Fisher Scientific, Inchinnan, UK) where absorbance ratios 260/230 and 260/280 were close to 2.

### 4.6. qPCR

Total RNA (1 µg) was reversely transcribed using the High-Capacity RNA-to-cDNA kit (Cat. 4387406, Thermo Fisher Scientific, Inchinnan, UK) following manufacturer’s instructions. qPCR was conducted using Maxima SYBR Green (Cat. K0221, Thermo Fisher Scientific, Inchinnan, UK). Samples were pipetted into corresponding well templates using the automated Labcyte ECHO 525 Acoustic Liquid handler system (Beckman Coulter, CA, USA). qPCR was conducted in the Lightcycler 480 (Roche, Indianapolis, IN, USA). Each sample was measured three times, and all genes were normalized to the endogenous reference gene 18S and RPL11. mRNA expression levels of target genes were calculated as fold change.

### 4.7. Immunocytochemistry

RPE were fixed in 2% paraformaldehyde (Cat. 043368.9M, Thermo Fisher Scientific, Inchinnan, Scotland, UK) for a total of 20 min, rinsed in PBS, and blocked with 5% FBS (Cat. 10270106, Thermo Fisher Scientific, Inchinnan, UK) and permeabilized with 0.1% Triton X-100 (Cat. X100-100ML, Sigma-Aldrich, Gillingham, UK). Cells were then incubated overnight (4 °C) with their corresponding primary antibodies (Table 1) diluted in PBS, followed by incubating with fluorophore-conjugated secondary antibodies (Table 2) at room temperature for 1 h. Cells were cover-slipped with DAPI-Vectashield (Vector Labs, Burlingame, CA, USA) and examined by confocal microscope (SP8, Leica Ltd., Wetzlar, Germany).

### 4.8. Metabolic Assessments

Primary RPE were seeded at a density of 2.5 × 10^3^/100 µL in complete ECM media with treatment times and conditions described above. Three assays measuring four metabolite assessments were performed, including Glucose-Glo (Cat. J6021, Promega, Seattle, WA, USA), Lactate-Glo (Cat. J5021, Promega, Seattle, WA, USA), and Glutamine/Glutamate-Glo (Cat. J8021, Promega, Seattle, WA, USA). Cell supernatants were collected after four days of Nintedanib treatment in the presence of TGF-β2 media or for corresponding control conditions. Supernatants were diluted 100-fold with PBS as per the manufacturer’s instructions. The luminescence for each metabolite along with the assay standards prepared were measured using a plate reader. Metabolite concentrations were determined using the standard curve of assay standards.

### 4.9. Seahorse XFe96 Assay

RPE real-time metabolism was assessed using the Seahorse XFe96 analyzer (Agilent, Santa Clara, CA, USA). Primary RPE were seeded at a density of 2.5 × 10^3^ per 80 µL of complete ECM media with treatment times and conditions described above. The day before the assay, the 96-well sensor cartridge (Cat. 102601-100, Agilent, Santa Clara, CA, USA) was hydrated overnight at 37 °C with no CO_2,_ along with 25 mL of Calibrant. The assay media (Cat. 103575-100, Agilent, Santa Clara CA, USA) and injections were prepared as per the manufacturer’s instructions. Glycolysis Stress kit (Cat. 103020-100, Agilent, Santa Clara, CA, USA) and Mito Stress kit (Cat. 103015-100, Agilent, Santa Clara, CA, USA) were used for metabolic assessment. phRPE were fixed with 2% paraformaldehyde (PFA) for 20 min at RT and stained with DAPI. Each well was imaged using a Leica DMi8 epifluorescence microscope (Leica Microsystems Ltd., Buffalo Grove, IL, USA.) and nuclei numbers were used to normalize seahorse data. Seahorse Wave Controller Software 2.4 was used for analysis and report generator.

### 4.10. Animals

*C57BL/6J* mice between 2 and 4 months of age were utilized in this study. All animals were housed within a controlled experimental facility with a 12 h light/dark cycle and unrestricted access to food and water. All experimental procedures were conducted under the regulation and compliance with UK Home Office Animals (Scientific Procedures) Act 1986, (License number 2876, approved 28 October 2019. This study received ethical approval from the Animal Welfare and Ethical Review Body (AWERB) of Queen’s University Belfast and work was conducted in compliance with the Association for Research in Vision and Ophthalmology Statement for the Use of Animals in Ophthalmology and Vision Research.

### 4.11. Two-Stage Laser-Induced Subretinal Fibrosis

Subretinal fibrosis using a two-stage laser procedure was induced using a protocol previously described by our group [9]. In brief, induction of choroidal neovascularization (CNV) occurred through the use of the laser photocoagulator (HGM Medical Laser System Inc., Salt Lake City, UT, USA). A total of four laser spots per eye were delivered, at the 12, 3, 6, and 9 o’clock positions around the optic disk. The laser settings were set to a power of 250 mv, a pulse duration 0.1 s, and spot size 100 μm. After seven days, a second laser burn was administered to each CNV lesion using the same laser settings described.

### 4.12. Intravitreal Injection of Nintedanib

One microliter of Nintedanib suspension formulation (12 µg/μL/eye) was injected using a 33G needle immediately following the 2nd laser (day 0, D0). Control mice were injected with the same volume of vehicle (0.015% Tween 80 in PBS). Additional control groups including Aflibercept (recombinant anti-VEGF fusion protein cross-reactive to mouse VEGF; 40 mg/mL) and anti-TNP control antibody (20 mg/mL) were also included in a separate study. The animals received one microliter injection of their corresponding compound following the 2nd laser (D0). Ten days (D10) later, fundus photography and fluorescence angiography were performed. Mice eyes were enucleated and fixed in 2% paraformaldehyde (PFA) (Cat. 158127, Sigma-Aldrich, Gillingham, Dorest, UK) for 2 h and processed for RPE/choroid flat mount investigation.

### 4.13. Fundus Fluorescence Angiography

The Micron IV system (Phoenix Technology Group, Pleasanton, CA) was employed to perform fundus imaging and fundus fluorescein angiography (FFA). The FFA was carried out 5 min after intra-peritoneal injection of 100 μL of 10% sodium fluorescein (Cat. F6377, Sigma-Aldrich, Gillingham, Dorest, UK).

### 4.14. Immunohistochemistry

RPE/choroidal flat mounts were stained according to previously published protocols [9,15]. The primary and secondary antibodies utilized in the study are listed in Table 1 and Table 2, respectively. RPE/choroidal flat mounts were then cover-slipped with Vectashield (Vector Labs, Burlingame, CA, USA) and samples were examined by Leica DMi8 epifluorescence microscope (Leica Microsystems Ltd., Wetzlar, Germany). Quantification of the fibrotic lesions in RPE/choroid flat mounts followed a previously established methodology [9]. All measurements were performed in a double blinded manner by independent researchers.

### 4.15. Data Analysis

GraphPad Prism (V11, GraphPad Software, San Diego, CA, USA) was used for statistical analysis of data. Data were presented as mean ± SD. A two-tailed paired or unpaired *t*-test was used to compare two groups, depending on experimental modal design. A one-way ANOVA with Tukey’s post hoc analysis was used when more than two groups were compared. Statistically significant values were set at *p*-value < 0.05.

## Figures and Tables

**Table 1:** Recombinant proteins and primary antibodies used in the study.

**Recombinants/Molecules**			
	Cat. No.	Company	
Recombinant Mouse Transforming Growth Factor Beta 2 (TGF-β2)	7346-B2-005	R&D Systems, Abingdon, Oxfordshire, UK	
**Primary Antibodies**			
	Cat. No.	Company	Host
CoraLite^®^ Plus 488-conjugated ZO-1 Polyclonal antibody	CL488-21773	Proteintech, Manchester, UK	rabbit
CoraLite^®^555-conjugated Vimentin Recombinant antibody	CL555-80232	Proteintech, Manchester, UK	rabbit
CoraLite^®^ Plus 647-conjugated smooth muscle actin specific Monoclonal antibody	CL647-67735	Proteintech, Manchester, UK	rabbit
CoraLite^®^594-conjugated Fibronectin Polyclonal antibody	CL594-15613	Proteintech, Manchester, UK	rabbit
Anti-Collagen Type I Antibody	AB758	Sigma-Aldrich, Gillingham, Dorest, UK	Goat
Griffonia Simplicifolia Lectin I (GSL I) Isolectin B4, Biotinylated	B-1205-5	Vector Laboratories, Newark, CA, USA	

**Table 2:** Secondary antibodies used in the study.

Secondary Antibodies		
	Cat. No.	Company
Alexa Fluor^®^ 488 AffiniPure™ Donkey Anti-Goat IgG (H+L)	705-545-147	Jackson ImmunoResearch, Ely, Cambridgeshire, UK
Alexa Fluor^®^ 594 Streptavidin	016-580-084	Jackson ImmunoResearch, Ely, Cambridgeshire, UK

## Data Availability

The data and material that support the findings of this study are available from the corresponding author on reasonable request.

## Supplementary Materials

The following supporting information can be downloaded at: https://www.mdpi.com/article/10.3390/ijms26157131/s1.

## Abbreviations

The following abbreviations are used in this manuscript:

ARPE-19	Adult retinal pigment epithelial cell line
AMD	Age-related macular degeneration
ANOVA	Analysis of variance
CNV	Choroidal neovascularization
CTGF	Connective tissue growth factor
DAPI	4′,6-Diamidino-2-phenylindole
DMEM	Dulbecco’s Modified Eagle medium
ECAR	Extracellular acidification rate
ECM	Extracellular matrix
EMT	Epithelial–mesenchymal transition
FBS	Fetal bovine serum
FGF	Fibroblast growth factor
FFA	Fundus fluorescein angiography
FGFR	Fibroblast growth factor receptor
FN	Fibronectin
GA	Geographic atrophy
GLUT1	Glucose transporter 1
HK2	Hexokinase 2
ICAM-1	Intercellular adhesion molecule-1
IPF	Idiopathic pulmonary fibrosis
LDHA	lactate dehydrogenase A
LDHB	lactate dehydrogenase B
MET	Mesenchymal–epithelial transition
nAMD	Neovascular age related macular degeneration
OCR	Oxygen consumption rate
P	Passages
PBS	Phosphate-buffered saline
PCR	Polymerase chain reaction
PDGF	Platelet derived growth factor
phRPE	Primary human retinal pigment epithelial cells
RPE	Retinal pigment epithelial cells
RT	Room temperature
PDGFR	Platelet derived growth factor receptor
PVR	proliferative vitreoretinopathy
RTKs	Receptor tyrosine kinases
SSc-ILD	Systemic sclerosis-associated interstitial lung disease
TCA	Tricarboxylic acid
TKI	Tyrosine kinase inhibitor
VEGF	Vascular endothelial growth factor;
VEGFR	Vascular endothelial growth factor receptor
αSMA	alpha-smooth muscle actin
TNF-α	Tumor necrosis factor alpha
TGF-β	Transforming growth factor beta
PFKFB3	6-phosphofructo-2-kinase/fructose-2,6-bisphosphatase 3
FCCP	Carbonyl cyanide-4 (trifluoromethoxy) phenylhydrazone
