# Supplementary material for: Nintedanib Induces Mesenchymal-to-Epithelial Transition and Reduces Subretinal Fibrosis Through Metabolic Reprogramming
Source: Int J Mol Sci. 2025 Jul 24;26(15):7131. doi: 10.3390/ijms26157131 (PMC12346381; doi:10.3390/ijms26157131)
Supplement: Supplementary file 1 [file ijms-26-07131-s001.zip › ijms-3745824-supplementary.pdf]

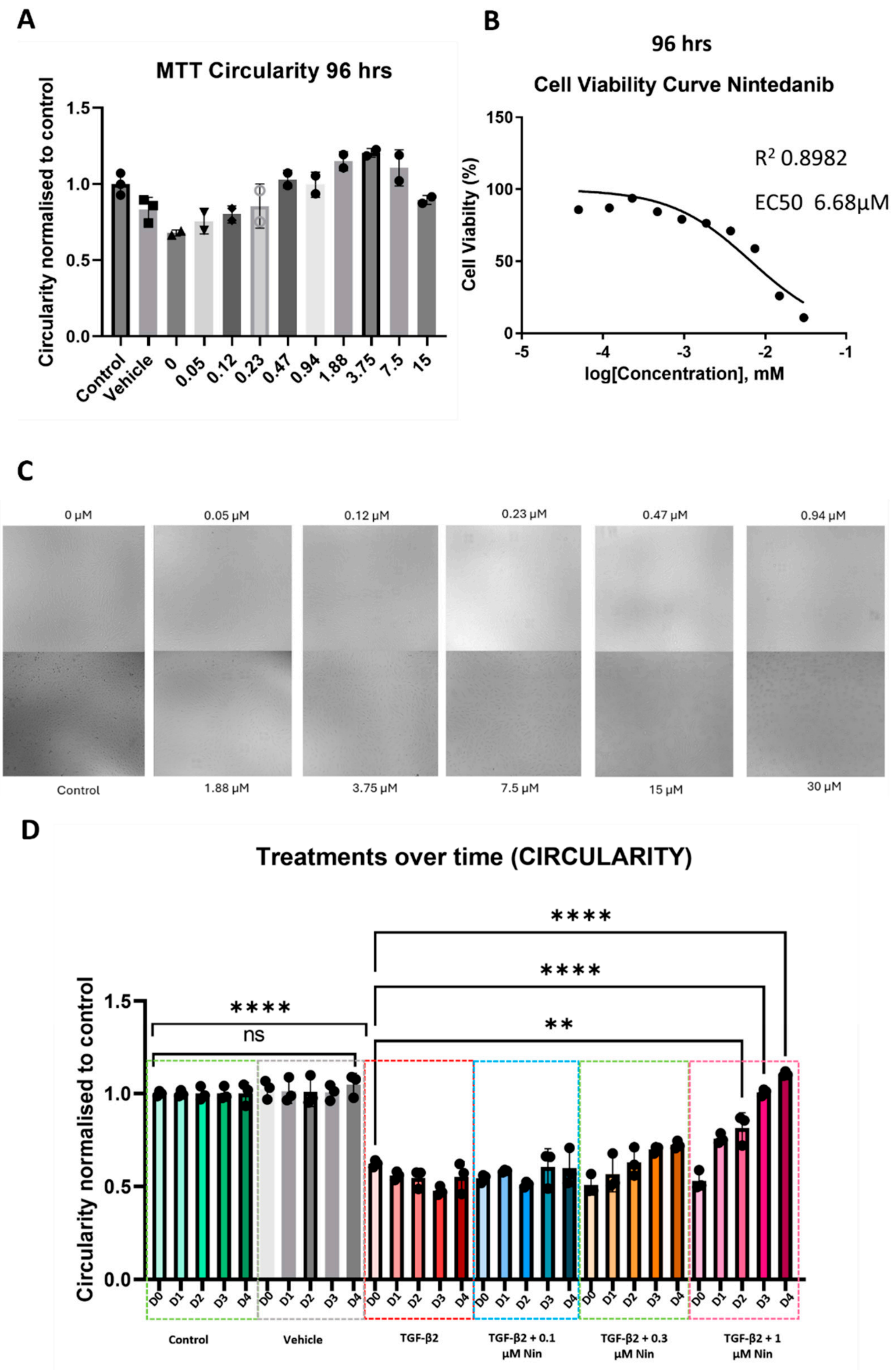

Supplementary Figure S1. Optimization of Nintedanib concentration and cell viability. Adult retinal pigment epithelial cell line (ARPE-19) cells were exposed to varying concentrations of Nintedanib post treatment of transforming growth factor beta 2 (TGF-β2) for 48 hrs. Nintedanib treatment was administered and left for a period of 96 hrs before morphological (A) and viability using an MTT assay (B) assessments were made. (A) Morphological assessment at 96 hrs were carried

out with ImageJ. Data is representative of 2-3 repeated studies and normalized to control groups. (B) Quantitative assessment of ARPE-19 cell viability using MTT assessment after Nintedanib exposure for 96 hrs. Data is representative 2-3 repeated studies and shown as cell viability %. (C) Representative images for each concentration of Nintedanib assessed after 96 hrs of exposure. (D) Morphological assessment of different Nintedanib concentrations over 4 days (96 hrs), Data is representative of 3 repeated studies and normalized to control groups. ns, not significant; ** $p<0.01$; **** $p<0.0001$. One-way ANOVA, with Tukey's post-hoc analysis was used.

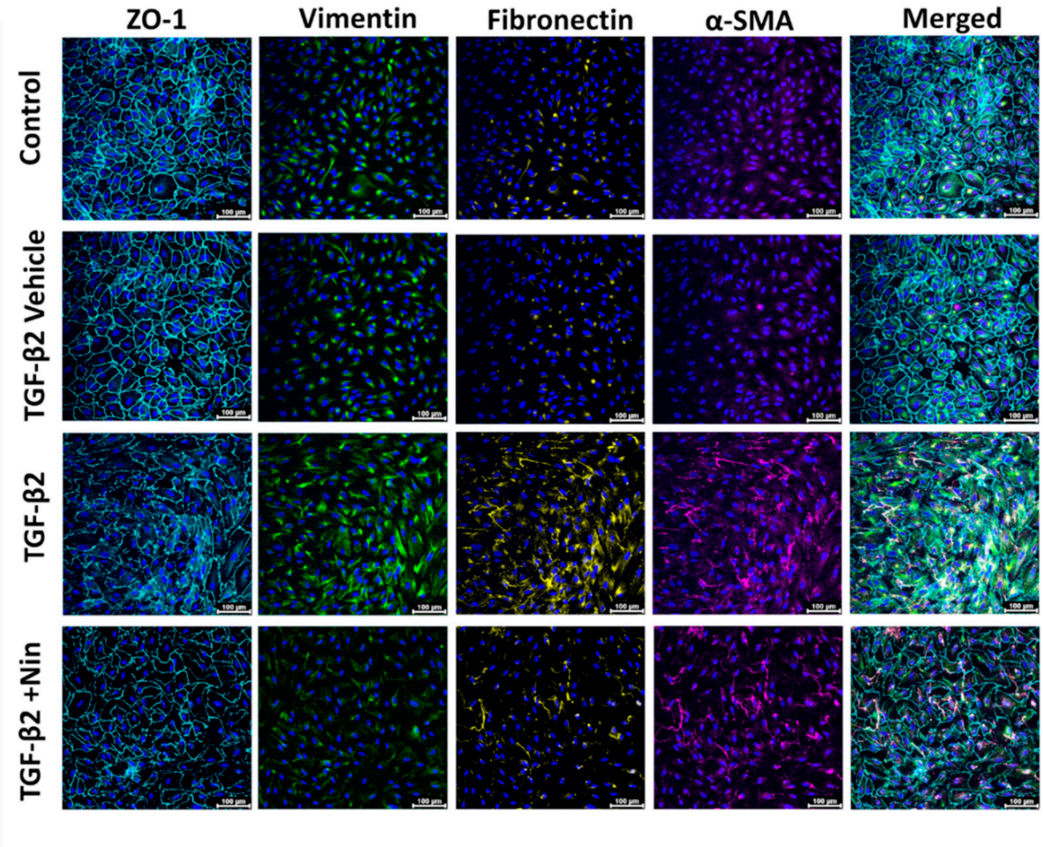

Supplementary Figure S2. Effect of Nintedanib on transforming growth factor beta 2 (TGF-β2) induced epithelial-to-mesenchymal transition in adult retinal pigment epithelial cell line (ARPE-19) cells. Representative immunocytochemistry images for zonula occludin-1 (ZO-1), vimentin, fibronectin, and alpha-smooth muscle actin (αSMA) in different groups. Scale bar 100 μm. Blue; Dapi; Cyan; ZO-1, Green; Vimentin; Yellow; Fibronectin, Red; α-SMA.

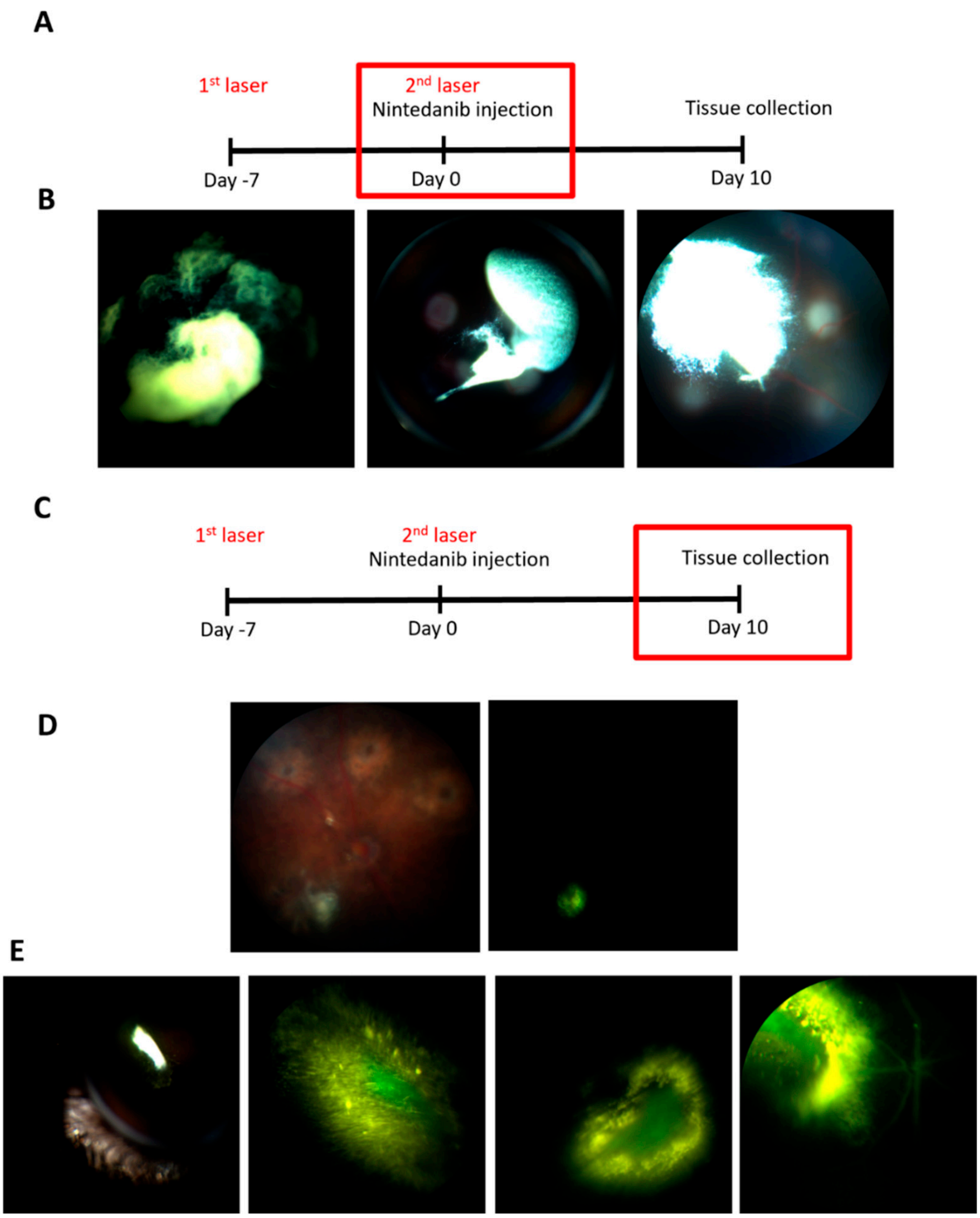

Supplementary Figure S3. Nintedanib detectable and remains present at and up to 10 days post injection in a mouse model of subretinal fibrosis. (A) schematic of the subretinal fibrosis *in vivo model* experimental design, highlighting (red box) when Nintedanib (12mg/ml) is injected intravitreally immediately following the second laser day 0. (B) Representative fundus images of Nintedanib present in the vitreous of the eye following immediate injection after the second laser day 0. (C) Schematic of the subretinal fibrosis *in vivo* model experimental design, highlighting (red box) day 10 where mice received fundus imaging and fluorescence angiography before tissue collection occurred. (D, E) Representative fundus and fluorescence angiography images of lesions and Nintedanib present at the site of lesions and visibly present within the vitreous of the mice eyes (yellow deposits).

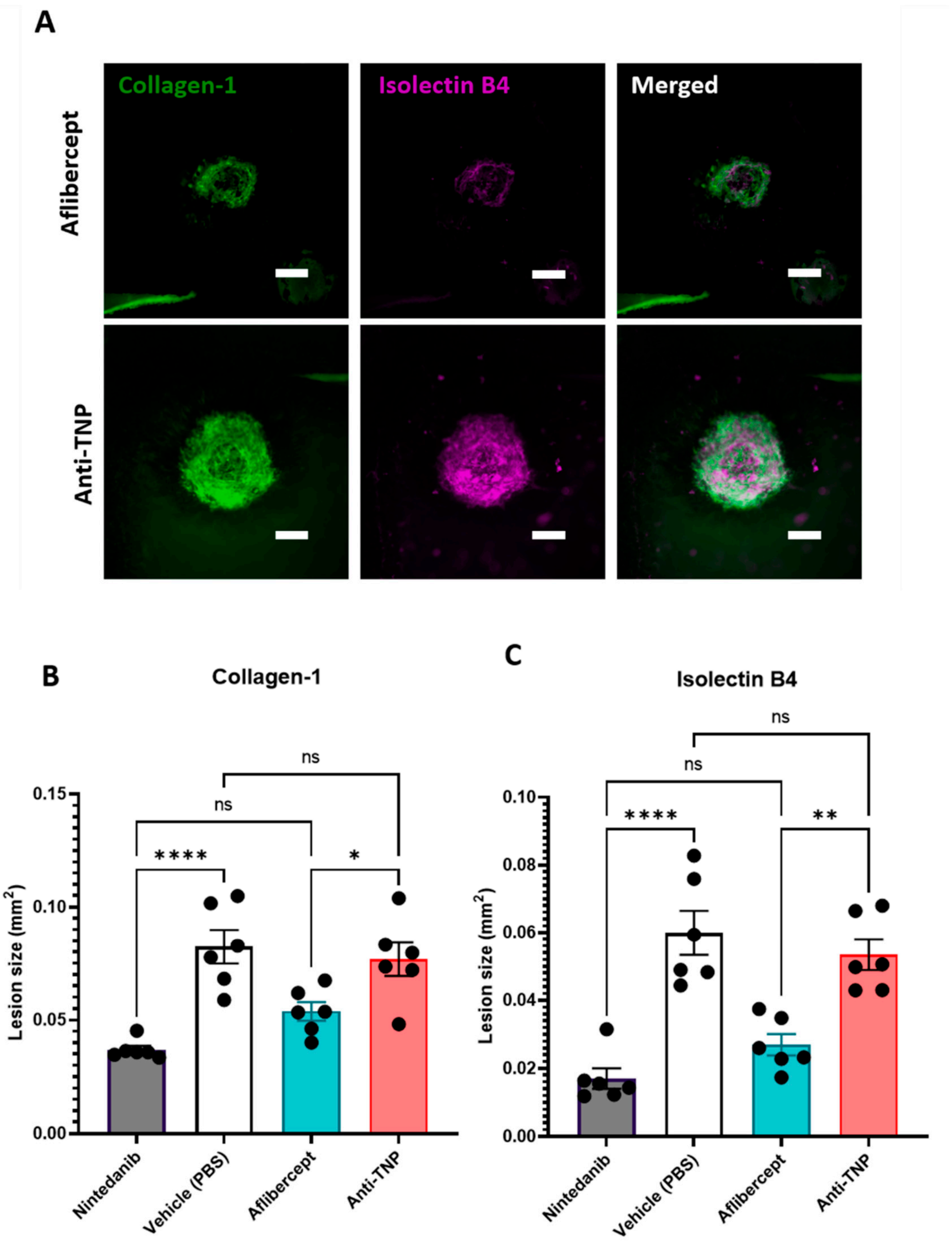

Supplementary Figure S4. Effect of Aflibercept and anti-TNP control antibody on a mouse model of subretinal fibrosis. (A) Representative lesion area respectively in Aflibercept and anti-TNP treated mice at day 10 post-second laser. Scale bar 100 µm. (B) Quantitative assessment of collagen-1+ (C) and Isolectin B4+ lesion area. Individual data points and mean ± SD are shown. n = 6 animals per group, (each point represents 8 lesions across two eyes analyzed). * $p < 0.05$; ** $p < 0.01$; **** $p < 0.0001$. One-way ANOVA, with Tukey's post-hoc was used for analysis.
